# Pitfalls in the diagnosis of a tumefactive demyelinating lesion: A case report

**DOI:** 10.1186/1752-1947-5-217

**Published:** 2011-06-07

**Authors:** Maria Gavra, Efstathios Boviatsis, Lampis C Stavrinou, Damianos Sakas

**Affiliations:** 1 Department of CT and MRI, Children's Hospital, "Agia Sophia'', Thivon and Papadiamantopoulou Street, Athens, Greece; 2 Department of Neurosurgery, University of Athens Medical School, "Evangelismos" General Hospital, 45-47 Ipsilantou Street 10676, Athens, Greece

## Abstract

**Introduction:**

In rare instances, demyelinating disorders manifest as tumefactive lesions that simulate brain tumors. We report a patient with a space-occupying lesion in the parietal lobe, which presented a serious diagnostic dilemma, between a rare tumefactive demyelinating disease, such as Balo concentric sclerosis and a glioma. This case report highlights important diagnostic clues in the differential diagnosis of Balo concentric sclerosis.

**Case presentation:**

A 20-year-old Caucasian woman with acute onset of left-sided weakness and numbness was admitted to hospital with neurologic signs of left-sided hemiparesis and hypoesthesia. Brain magnetic resonance imaging showed a mass lesion of abnormal signal intensity with concentric enhancing rings in the right parietal lobe, without perifocal edema. The characteristic concentric pattern detected on the magnetic resonance images was highly suggestive of Balo disease, and corticosteroids were administered. Evoked potentials, cerebrospinal fluid analysis, and magnetic spectroscopy findings were not specific, and glioma was also included in the differential diagnosis. A stereotactic biopsy was not diagnostic.

After one month the patient showed moderate clinical improvement, and during 12 months follow-up, no further relapses occurred. In the follow-up magnetic resonance imaging, the concentric pattern had completely disappeared, and only a low-signal, gliotic lesion remained.

**Conclusion:**

We hope this case presentation will advance our understanding of clinical and radiologic appearance of Balo concentric sclerosis, which is a rare demyelinating disease. Although this is a specific entity, it has a broader clinical impact across medicine, because it must be differentiated from other space-occupying lesions in the central nervous system.

## Introduction

Tumefactive demyelinating brain lesions present a diagnostic challenge, because their clinical, radiologic, and even histologic features may complicate the identification of their true nature. This often leads to invasive and costly procedures, which frequently yield non-diagnostic results. We report a patient with a right parietal white matter lesion, who presented a serious diagnostic dilemma, as the lesion was difficult to differentiate between a rare demyelinating disease such as Balo concentric sclerosis (BCS) and a glioma. The characteristic magnetic resonance findings of the case, its acute onset, and its clinical improvement after corticosteroid therapy finally set the diagnosis of BCS. The risks of the stereotactic procedures that led to the misdiagnosis of BCS are discussed.

## Case presentation

**Figure 1:**
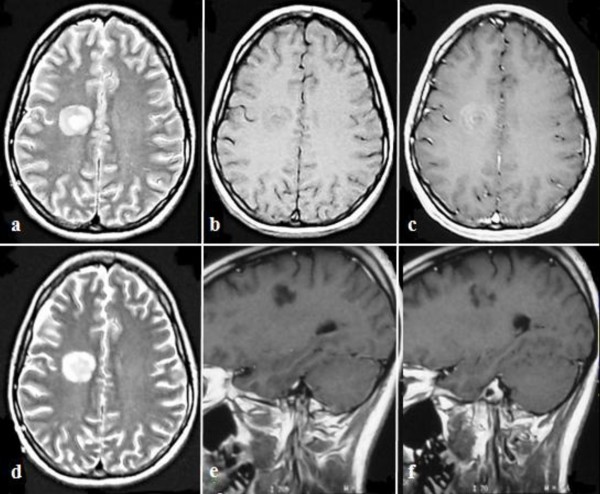
**Brain MR images in a 20-year-old woman**. **(a) **Axial T_2_-weighted image shows a hyperintense mass with concentric pattern in the right centrum semiovale. **(b) **Axial T_1_-weighted image reveals hypoisointense concentric rings in the white matter of the right parietal lobe. **(c) **Axial T_1_-weighted image after contrast shows concentric enhancing rings. **(d) **Axial T_2_-weighted image, 1 month after therapy, shows differences with decrease of the signal intensity at the center of the lesion. **(e, f) **Coronal T_1_-weighted image after administration of gadolinium demonstrates a low-signal, non-enhancing lesion.

A 20-year-old Caucasian woman, with no past medical history, presented to the emergency room of a general hospital, with numbness and weakness of her left-sided limbs. Neurologic examination revealed no cranial nerve deficit and 4/5 left-sided hemiparesis. No cerebellar impairment was noted. She was unable to localize tactile stimuli or to judge objects' size and shape. She had no pain, pressure, or temperature loss. Brain computed tomography (CT) demonstrated a large (2.1 cm), well-demarcated hypodense lesion in the right parietal lobe, without perifocal edema. Magnetic resonance imaging (MRI) without contrast showed a hypoisointense concentric mass on T_1_- and hyperintense on T_2_-weighted images (Figure 1a, b). After contrast, the lesion appeared to enhance inhomogeneously, in a pattern resembling separate, alternating enhancing rings (Figure 1c). These MRI findings were highly suggestive of the concentric pattern of demyelination (BCS).

Somatosensory evoked potentials (SSEPs), serum, and cerebrospinal fluid analysis were normal. Human immunodeficiency virus (HIV) and antinuclear antibody (ANA) tests were negative. The chest radiograph (CXR) was normal. Under the presumptive diagnosis of BCS, the patient received high-dose intravenously administered methylprednisolone (500 mg/day for ten days). The subsequent proton MR spectroscopy (MRS) revealed reduction in *N*-acetylaspartate and an increase in choline, lipids, and lactate. The findings were not specific and were consistent either with an acute demyelinating lesion or with a low-grade glioma. Ten days later, the patient showed moderate clinical improvement and continued with oral steroid treatment.

A brain CT-guided stereotactic biopsy was scheduled to establish the diagnosis, as MRS and laboratory findings were not specific. Four specimens within and from the periphery of the lesion were taken. Histologic examination failed to show the presence of a significant number of histiocytes, foamy macrophages, or myelin loss that would otherwise be expected in Balo sclerosis. It showed, however, mild to moderate nuclear atypia, whereas Ki-67 immunostaining was positive in 1% to 2% of the nuclei. The pathologist commented that the findings were suggestive of a grade II astrocytoma.

In the face of this diagnostic dilemma, a conservative approach was adopted. Oral steroid treatment was continued, and the patient was scheduled for a new 1H-MRS and MRI scan one month later. The spectroscopic findings were identical to the previous ones. However, the new conventional MRI images showed significant differences, in that the signal intensity was lower in the center of the lesion in T_2 _images (Figure 1d), whereas the enhancing rings appeared to fade away centrifugally (Figure 1e, f). The dimensions of the lesion were unchanged. These findings were considered sufficient enough to establish the diagnosis of BCS.

No relapse in the symptoms occurred during the next 12 months of follow-up. A serial MRI showed a low-signal, non-enhancing lesion.

## Discussion

Diagnosis of tumefactive brain lesions is challenging to both clinicians and radiologists. Clinical differential diagnosis includes demyelinating diseases, neoplasms, and infections such as abscesses. Such lesions with mass-like characteristics may be the presenting feature of multiple sclerosis (MS), acute disseminated encephalomyelitis, or other rare demyelinating diseases, such as BCS and Marburg type. BCS is a rare demyelinating disease considered to be an acute variant of MS, appearing in young adults and typically following a fulminant course [1]. It shows a monophasic, rapidly progressive course, sometimes fatal. Histologically, it is characterized by a large lesion consisting of rings of demyelination alternating with rings of intact myelin. MRI is the method of choice for imaging demyelination lesions, tumefactive or not. Although the usual appearance of MS is that of multiple, small, demyelinating plaques, in some cases, it can simulate a mass lesion, which it would be hard to distinguish from a brain tumor [2]. MRI characteristics, such as open-ring enhancement, peripheral restriction on diffusion-weighted imaging, or venular enhancement, may be rewarding in differentiating tumefactive MS lesions from neoplastic ones [2]. BCS is also considered within the spectrum of MS. It shares an apparent basic pathologic similarity to MS, with the exception of a lamellar pattern. The striking concentric pattern of demyelination distinguishes this disorder from other demyelinating diseases. BCS has characteristic MRI features such as the hypoisointense concentric rings on T_1_-weighted, the whirlpool hyperintense concentric rings on T_2_-weighted, and the separate rings of enhancement in a concentric pattern [3]. This type of concentric pattern has not been described in association with any other demyelinating/inflammatory diseases except BCS, and therefore, acute disseminated encephalomyelitis and Marburg MS were excluded in our case.

Advanced neuroimaging can provide important *in vivo *markers of disease progression. MRS in BCS may show reduction of *N*-acetylaspartate and increase in choline and lipids, reflecting axonal destruction and an elevation of lactate resonance due to local ischemia from the ongoing inflammatory process [4]. These resonance spectra [2,4] are not specific for BCS, and they may resemble those of brain tumors and acute MS plaques [2,4,5]. The chronic demyelinating plaque, however, shows a completely different pattern [4].

A stereotactic biopsy and histologic examination of the lesion is the final diagnostic approach in equivocal cases [6]. It is safe and reliable, especially if specimens from multiple sites within the lesion are targeted. It has a diagnostic accuracy of 82% to 99% [7]. Acute demyelinating plaque is hypercellular, and on frozen sections, this hypercellularity may be mistaken to be indicative of glioma. The diffuse infiltration of inflammatory cells, mainly reactive astrocytes and lipid-laden macrophages, and perivascular cuffing by T-lymphocytes favors the diagnosis of demyelinating plaque. The presence of alternating rings of myelin preservation or remyelination and myelin loss, consistent with demyelination, corresponds to the concentric type of demyelination, or BCS. Although the presence of reactive astrocytes can raise the diagnosis of an astrocytoma, in the non-neoplastic demyelinating plaque, these astrocytes are not significant in number to establish such a diagnosis safely, nor are there areas of vascular proliferation, indicative of a neoplastic process. Staining for myelin and axons and applying special immunohistochemical stains for macrophage markers should help overcome this diagnostic pitfall [8].

Occasionally, the biopsy is non-diagnostic or dispensable [5]. In our case, the risk lay in the fact that in BCS, areas of demyelination alternate with areas of active gliosis in a dynamic and concentric fashion. Even with the use of stereotactic procedures, tissue specimens may be yielded from area of reactive gliosis not just outside, but also from within the lesion itself, thus giving ambiguous or false results. Targeting multiple areas within the lesion may help overcome this problem.

In our case, histopathologic findings from the biopsy were misleading.

However, the characteristic concentric rings of demyelination alternating with myelination on MRI, the patient's considerable clinical improvement after steroid therapy, and the signal differences in follow-up MRI scans established the diagnosis of BCS.

In serial MRI scans, the concentric ring enhancement of BCS is expected to fade away centrifugally, until it appears as a low-signal, non-enhancing lesion, typical of a chronic demyelinating plaque [9], as in our case. Regarding MRS, it seems that it is the serial changes of the metabolites' resonance intensities rather than the individual values that provide more information about the nature of the lesion [9].

## Conclusion

Demyelinating diseases can mimic brain neoplasms clinically, radiologically, and histopathologically. BCS is a rare demyelinating disease, which can manifest as a mass lesion. The typical concentric pattern on MR images, along with clinical features, can lead to accurate diagnosis and treatment. For suspected cases, it is advisable to use steroid therapy or undergo serial MRI examinations. However, in borderline cases, pathologic evidence is beneficial to a final diagnosis.

## Abbreviations

BCS: Balo concentric sclerosis; CT: computed tomography; MRI: magnetic resonance imaging; MRS: proton magnetic spectroscopy; MS: multiple sclerosis.

## Competing interests

The authors declare that they have no competing interests.

## Consent

Written informed consent was obtained from the patient for publication of this case report and any accompanying images. A copy of the written consent is available for review by the Editor-in-Chief of this journal.

## Authors' contributions

MG collected and analyzed all patient data, conducted a literature review, and was a major contributor in writing the manuscript. LS collected and analyzed data related to the patient's stay in the neurosurgery department and collected the follow-up information. EB and DS provided clinical details and technical input, revised the manuscript, and performed changes throughout the manuscript. All authors read and approved the final manuscript.
